# *Helicobacter pylori* infection among the Dayak community in Kuching Division, Sarawak: findings from a community-based study

**DOI:** 10.1186/s12876-026-04947-7

**Published:** 2026-05-19

**Authors:** Whye Lian Cheah, Sohail Mushtaq, Sze Kiat Sim, Christina Busak Henry Sum Agong

**Affiliations:** Faculty of medicine and health Sciences, Universiti Malaysia Sarawak, Kota Samarahan, Malaysia

**Keywords:** *Helicobacter pylori*, Rural communities, Food frequency Questionnaire

## Abstract

**Purpose:**

*Helicobacter pylori* is a primary driver of peptic ulcer disease and gastric adenocarcinoma, yet epidemiological data regarding its prevalence among indigenous communities in Sarawak are scarce. This study aimed to determine the *H. pylori* seroprevalence and identify associated socio-demographic and lifestyle factors within the Dayak population.

**Method:**

It was a cross-sectional study carried out in rural areas of Kuching Division. Data was collected using blood sampling, interview-guided questionnaires, and anthropometric measurements. The questionnaire included socio-demographic profiles, history of illness, smoking habit, alcohol consumption, physical activity, and dietary intake. Factor analysis was used to identify dietary patterns. Health profile including *helicobacter pylori* infection status, blood pressure and body mass index were collected.

**Result:**

A total of 199 respondents aged 18 to 91 years from six villages participated in this study, yielding a response rate of 96.6%. About 60% of the respondents were found to be overweight or obese, and 48.7% had hypertension. Blood test analysis detected *H. pylori* antibodies in 46.2% of the respondents. In the multivariate analysis, several factors were found to be significantly associated with the presence of *H. pylori* antibodies: increasing age (OR: 1.042, 95% CI: 1.011–1.074), lower systolic blood pressure (OR: 0.967, 95% CI: 0.941–0.994), high physical activity level (OR: 0.278, 95% CI: 0.121–0.640), and medium intake of nutritious food and dairy (OR: 3.364, 95% CI: 1.394–8.117).

**Conclusion:**

Implementing community-level screening programs is essential for early detection and targeted intervention, which could ultimately reduce the burden of infection and its associated complications.

## Introduction

*Helicobacter pylori* infection is one of the most common infections worldwide, currently affecting an estimated 43.1% of the global population [1]. While prevalence is decreasing, a systematic review by Namikawa et al. [2] highlights significant regional variation; for instance, prevalence remains high in Mongolia (74%) compared to Switzerland (19%) and the US (36%) [3, 4]. A notable trend is the marked decline in East Asia, particularly among younger populations. In Japan, infection rates have dropped from 67% in the 1930 birth cohort to approximately 7% in those born in 2000, with a 15% decrease in risk for every successive 10-year cohort [5, 6, 7]. Despite these declining trends, the fact remains that most infected individuals remain asymptomatic, complicating efforts for early detection and treatment. While nearly all infected individuals exhibit gastric mucosal inflammation, it is estimated that 25–30% will eventually develop gastrointestinal or extra-gastrointestinal complications during their lifetime [8, 9, 10]. *H. pylori* has been associated with peptic ulcer disease and gastric cancer [11, 12]. Several epidemiological studies have shown that the incidence of gastric cancer is high in Asian countries such as China, Japan, and India [13].

In Malaysia, the seroprevalence of *H. pylori* has been reported to range from 10% to 55%, with the highest rates observed among the Chinese (40–50%) and Indian (50–55%) populations [14]. Epidemiological studies conducted in East Malaysia have reported that the indigenous ethnic groups of Sabah and Sarawak have seroprevalence rates as high as 65.3% and 55.5%, respectively [15]. However, a study among the Penan indigenous group in Sarawak showed a lower prevalence *H.plyori* infection of 37.5% [16].

Studies have shown that *H. pylori* is mainly transmitted through oral-oral and fecal-oral contact, as well as through contaminated water sources [17, 18]. Additionally, epidemiological studies have linked *H. pylori* infection to the development of obesity, metabolic syndrome, and cardiovascular disease [19, 20, 21]. As *H. pylori* is related to the development of peptic ulcer disease, factors such as smoking, alcohol consumption, and physical inactivity have been identified as indirect risk factors for infection [22, 23]. Reports on gastric carcinoma incidence suggest that *H. pylori* infection can lead to gastric cancer later in life, and that lifestyle choices and dietary habits can increase the risk of infection [24, 25]. Consuming pickled, fermented, smoked, or salty foods, as well as diets high in carbohydrates and simple sugars, has been found to be associated with an increased risk of *H. pylori* infection [26, 27]. Xia et al. [26] found that a high-protein dietary pattern—including animal offal, animal blood, fish, seafood, and poultry—as well as a grains-vegetables pattern, were associated with a reduced prevalence of *H. pylori* infection. Similarly, Shu et al. [27] reported that prudent and traditional dietary patterns, rich in vegetables, fruits, soy products, fish, rice, and wheat, were linked to a lower risk of *H. pylori* infection.

While most published data on *H. pylori* infections come from hospital settings, implementing community-level screening can offer significant advantages. Early identification of infections within the community allows for timely treatment, prevention of complications, and control of infection spread. This proactive approach supports more effective preventive healthcare strategies. Additionally, it enables the identification of individuals or communities at higher risk of *H. pylori* infection, allowing for targeted interventions. Infected individuals can receive eradication therapy, and those exhibiting red flag symptoms can be referred to hospitals for further management. Such measures play a crucial role in the primary prevention of *H. pylori*-associated conditions, including peptic ulcer disease, upper gastrointestinal bleeding, perforated peptic ulcers, chronic gastritis, and, importantly, gastric cancer.

Previous studies [15, 16] have only reported on segments of the population in Sarawak and did not explore the most predominant indigenous group—the Dayak communities. Given that the Dayak represent the largest indigenous group in Sarawak, understanding the prevalence and impact of *H. pylori* infection within this population is crucial. The unique cultural practices, dietary habits, and genetic backgrounds of the Dayak may influence both the transmission and clinical outcomes of *H. pylori* infection. The traditional longhouse architecture involves high-density communal living in shared spaces (the *ruai*) which, when combined with a reliance on untreated gravity-feed water systems and the frequent consumption of traditional fermented foods such as *kasam*, creates a distinct environment that facilitates both person-to-person and waterborne transmission of *H. pylori* [28]. However, there is currently a lack of data specifically addressing this group. Therefore, this exploratory study aimed to determine the seroprevalence of *H. pylori* (primary outcome) and identify its associated socio-demographic and lifestyle factors (secondary outcomes) among the Dayak communities of Sarawak. By filling this critical knowledge gap, the findings provide a foundation for targeted public health strategies and a more comprehensive understanding of indigenous gastric health.

## Method

This was a cross-sectional study carried out among Dayak communities in the Kuching Division. The Kuching Division covers an area of approximately 4,559 square kilometers and is one of the larger administrative divisions in Sarawak. It has an estimated population of over 700,000 people, with the Bidayuh and Iban—the most predominant indigenous groups—comprising the Dayak communities. These communities have a significant presence in the rural areas surrounding Kuching Division, especially in the Bau and Lundu districts. The list of localities with the most populated Dayak communities was obtained with permission from the respective district offices. Based on this list, a total of 10 villages were randomly selected.

The sample size for this study was determined using the formula n = (Z² × P × (1 - P)) / e². In this formula, Z represents the value from the standard normal distribution corresponding to the desired confidence level (Z = 1.96 for a 95% confidence interval). The variable P denotes the expected true proportion, while e signifies the desired precision, defined as half the width of the desired confidence interval. Given a prevalence rate of 37.5% [16], a precision margin of error of 7%, the minimum sample size required for this study was calculated to be 200, with an attrition rate of 5%. A total of 199 respondents participated in the study (99.5% response rate). This minor deviation is not expected to impact the statistical significance or the validity of the associations observed.

The inclusion criteria for the study were as follows: participants must be Dayak population, aged 18 years and above, and residing in the Kuching Division. Additionally, they had to be able and willing to participate in the study. The exclusion criteria included individuals with severe cognitive impairment, those with major medical illnesses requiring immediate medical attention, and individuals who could not communicate in English or Malay.

After approaching the villages, only six village heads gave consent to carry out the study due to scheduling conflicts with ongoing community events and celebrations. Announcements were made during social activities and via village social media. On the day of data collection, villagers gathered at the community hall or church compound. Written consent was obtained prior to data collection on the same day.

Data collection methods included blood sampling, interview-guided questionnaires, and anthropometric measurements. Blood samples were collected under aseptic conditions via venipuncture from the antecubital vein into sterile plain tubes. To ensure logistical feasibility and participant compliance in a community-based setting, serological testing for *H. pylori*-specific IgG antibodies was utilized. While immunochromatographic stool antigen tests can identify active infection, serology was selected as the most practical modality for this study due to the challenges of maintaining a cold chain for fecal samples during transport from remote rural locations to the laboratory. Furthermore, while serology cannot distinguish between an active infection and a past exposure, it remains a highly validated, minimally invasive, and cost-effective tool for assessing the cumulative prevalence of exposure within a population—the primary objective of this study. The samples were processed and analyzed at a certified private laboratory using a standardized immunoassay (ELISA) to ensure high sensitivity and specificity.

The questionnaire included the collection of socio-demographic profiles and history of illness, including history of gastric problems. Dietary intake was assessed using a modified version of the food frequency questionnaire (FFQ) from the Malaysian Adults Nutrition Survey (MANS 2014) [29]. While this tool was originally validated for the general Malaysian population and not specifically for indigenous Dayak groups, it was selected as the most comprehensive standardized tool available to capture general dietary trends across all Malaysian ethnicities. Respondents were asked about their consumption of various foods on a daily, weekly, or monthly basis.

Information on smoking habits was gathered to examine the smoking behavior of the respondents. Alcohol consumption was assessed using the Alcohol Use Disorders Identification Test (AUDIT), a 10-item screening tool developed by the World Health Organization (WHO) to evaluate alcohol consumption, drinking behaviors, and alcohol-related issues.

Blood pressure was measured using a digital blood pressure monitor, calibrated with a manual sphygmomanometer. Respondents were seated and relaxed for at least 5 min before measurement. Two readings were taken; if the initial reading was high, another reading was taken after 30 min. Classification of hypertension was based on the Clinical Practice Guidelines for the Management of Hypertension (5th edition, 2018), Ministry of Health Malaysia.

Physical activity level was assessed using the International Physical Activity Questionnaire (IPAQ). Respondents were asked to recall their physical activities over the last 7 days, reporting the frequency (days per week) and duration (minutes per day) of each type of activity. Physical activity levels were calculated in MET-minutes/week and categorized into low, moderate, and high physical activity. Height and weight were measured using a weighing scale and stadiometer, with two readings taken for consistency. Body Mass Index (BMI) was calculated using the formula: BMI (kg/m²) = Weight in kilograms / (Height in meters)². Classification of BMI was based on the Malaysian Clinical Practice Guidelines on the Management of Obesity (2004). Waist circumference was measured using a flexible, non-stretchable measuring tape.

Ethical approval for this study was obtained from the Medical and Ethics Committee of Universiti Malaysia Sarawak (UNIMAS/TNC(PI)/09–65/01 Jld.3(90)). All respondents were informed about the study and their right to withdraw. Confidentiality and anonymity were maintained by omitting personal information.

Data were entered and analyzed using IBM SPSS version 27.0. Descriptive data were generated. Normality testing was conducted to determine whether the dataset followed a normal distribution, which is crucial for validating the assumptions of both univariate and multivariate analyses. Multicollinearity diagnostics were performed using Variance Inflation Factor (VIF) and tolerance values to ensure that correlated anthropometric and cardiovascular variables did not destabilize the model; a VIF value > 10 was used to indicate high multicollinearity [30]. Before entering multiple logistic regression analysis, variables demonstrating a p-value of 0.25 and below in the univariate analysis were included as potential factors [30]. All determinations of statistical significance were based on a threshold of 0.05.

Dietary patterns were determined using principal component analysis (PCA). Data adequacy was assessed using the Kaiser-Meyer-Olkin (KMO) test. The data had to meet a KMO value greater than 0.5 and a Bartlett’s test of sphericity p-value < 0.05 [24] before proceeding to PCA. Varimax rotation was used to keep factors uncorrelated and to facilitate interpretability. A total of 17 food groups were retained. The number of factors to be extracted was determined based on the inflection point on the Scree plot and an eigenvalue > 1.0. The factors were then named based on the food groups with the highest factor loadings. Dietary patterns were determined using principal component analysis (PCA). A test of data adequacy using Kaiser-Meyer Olkin test measurement of sample was carried out. The data has to meet Kaiser-Meyer Olkin test greater than 0.5 and Bartlett test of sphericity of *p* < 0.05 [31] before moving to the PCA. Varimax rotation was used to keep factors uncorrelated and ease the interpretability between factors.

## Result

A total of 199 respondents aged 18 to 91 years from six villages participated in this study, yielding a response rate of 96.6%. The mean age was 55.2 years (SD = 14.42). The majority of respondents were Christians (97.5%), and more than two-thirds were married. Piped treated water was the most common source of water supply in the villages (72.4%). About 60% of the respondents were found to be overweight or obese, which is higher than the national prevalence of 54.4% reported in the National Health and Morbidity Survey (NHMS) 2019 [32], and 48.7% had hypertension, aligning with the prevalence of 30.0% to 50.0% often reported in rural Malaysian settings. Blood test analysis detected *H. pylori* antibodies in 46.2% of the respondents. More details on the socio-demographic characteristics, health profiles of the respondents, and their relationship with *H. pylori* infection are presented in Table 1.

**Table 1:** Socio-demographic characteristic and health profiles of the respondents (*N* = 199)

	Overall	*H.pylori*		*p* value
		Negative	Positive	
Age (year)	55.2 ± 14.42	53.5 ± 14.47	57.1 ± 14.17	0.074
Gender				
Male	80 (40.2)	41 (38.3)	39 (42.4)	0.559
Female	119 (59.8)	66 (61.7)	53 (57.6)	
Religion				
Christian	194 (97.5)	102 (95.3)	92 (100.0)	0.063
Islam	5 (2.5)	5 (4.7)	0 (0.0)	
Marital status				
Married	155 (77.9)	84 (78.5)	71 (77.2)	0.865
Not married/divorcee/ widow/widower	44 (22.1)	23 (21.5)	21 (22.8)	
Water source				
Piped treated water	144 (72.4)	100 (93.5)	84 (91.3)	0.600
Gravity feed	55 (27.6)	7 (6.5)	8 (8.7)	
Smoking				
Yes	44 (22.1)	21 (19.6)	23 (25.0)	0.395
No	155 (77.9)	86 (80.4)	69 (75.0)	
Occupation				
Not working/retired Student/housewife	116 (58.3)	58 (54.2)	58 (63.0)	0.249
Working	83 (41.7)	49 (45.8)	34 (37.0)	
Body mass index	26.9 ± 5.42	27.7 ± 5.84	25.9 ± 4.71	0.027
Normal	77 (38.7)	37 (34.6)	40 (43.5)	0.170
Overweight	71 (35.7)	37 (34.6)	34 (37.0)	
Obese	51 (25.6)	34 (30.8)	18 (19.6)	
Waist circumference (cm)	89.4 ± 15.59	91.8 ± 13.13	86.6 ± 17.70	0.019
Systolic blood pressure (mm/Hg)	133.5 ± 22.36	137 ± 18.21	129.3 ± 25.82	0.014
Diastolic blood pressure (mm/Hg)	79.9 ± 13.65	81.9 ± 11.46	77.5 ± 15.53	0.023
Blood pressure				
Normal	39 (19.6)	15 (14.0)	24 (26.1)	0.048
Elevated & hypertension	160 (80.4)	92 (86.0)	68 (73.9)	
Known illness				
Hypertension				
No	123 (61.8)	63 (58.9)	60 (65.2)	0.383
Yes	76 (38.2)	44 (41.1)	32 (34.8)	
Diabetes Mellitus				
No	175 (87.9)	97 (90.7)	78 (84.8)	0.275
Yes	24 (12.1)	10 (9.3)	14 (15.2)	
Heart Disease				
No	189 (95.0)	101 (94.4)	88 (95.7)	0.755
Yes	10 (5.0)	6 (5.6)	4 (4.3)	
Cancer				
No	198 (99.5)	107 (100.0)	91 (98.9)	0.462
Yes	1 (0.5)	0 (0.0)	1 (1.1)	
History of gastric				
No	125 (62.8)	62 (57.9)	63 (68.5)	0.143
Yes	74 (37.2)	45 (42.1)	29 (31.5)	
Physical activity				
Low	42 (21.1)	22 (20.6)	20 (21.7)	0.046
Medium	53 (26.6)	36 (33.6)	17 (18.5)	
High	104 (52.3)	49 (45.8)	55 (59.8)	
AUDIT (alcohol)				
Low risk	176 (88.4)	92 (86.8)	84 (91.3)	0.369
Increasing & high risk	21 (10.6)	14 (13.2)	8 (8.7)	

Table 2 presents the differences in food intake between respondents with positive and negative *H. pylori* serostatus. Univariate analysis showed that there was no significant relationship between food intake and *H. pylori* serostatus.

**Table 2:** Food intake and *Helicobacter pylori* serostatus (*N* = 199)

	Overall	H.Pylori		*p* value
		Negative	Positive	
Total Energy intake	1775.9 (IQR = 1023.58)	1739.4 (IQR = 905.02)	1818 (IQR = 1149.86)	0.588
Total Protein intake	76.4(IQR = 51.5)	75.6 (IQR = 48.24)	77.3 (IQR = 55.31)	0.813
Total Fat intake	44.2(IQR = 35.62)	41.9 (IQR = 28.06)	46.9 (IQR = 42.78)	0.322
Total Carbohydrate intake	263.4 (IQR = 140.04)	261.9 (IQR = 137.79)	265.1 (IQR = 143.36)	0.876
Total Fiber intake	6.1(IQR = 5.75)	6.4 (IQR = 5.86)	5.8 (IQR = 5.63)	0.474
Average amount of cooking oil used per month (kg)	3.1 ± 1.75	3.1 ± 1.86	3.2 ± 1.63	0.662
Average amount of sugar used per month (kg)	2.5 ± 1.6	2.4 ± 1.54	2.6 ± 1.68	0.295

After varimax rotation, factor analysis revealed four distinct dietary patterns and their corresponding factor loadings (see Table 3). These four dietary patterns accounted for 50.43% of the variance in total food intake. The Kaiser-Meyer-Olkin (KMO) measure of sampling adequacy was 0.644. Although this value exceeds the minimum acceptable threshold of 0.5, it is relatively low, suggesting that the items in the questionnaire do not correlate strongly enough to produce a highly robust factor structure. Therefore, these dietary patterns should be interpreted as exploratory trends rather than definitive dietary profiles. Additionally, Bartlett’s test of sphericity was significant (*p* < 0.001), further indicating the appropriateness of the analysis. To maintain the integrity of the factor loadings, a complete-case analysis approach was employed for this component; thus, PCA was conducted on the 181 participants who had complete responses across all dietary items, excluding 18 participants with partial missing data. The four dietary patterns were generated based on 17 food groups, which included fresh and dried mushrooms, salted and dried vegetables, bean sprouts, ladies’ fingers, other legumes, lollies, ice cream, cakes and pastries, salted eggs, sweet spreads, processed chicken and meat products, packaged snacks and kuih, burgers, fried chicken, pizza, fries, mashed potatoes, coleslaw, processed fish and seafood products, fruits, brown rice, cereals, grains, corn, yogurt, cheese, evaporated milk, and dried fruits. The first pattern, “plant-based food,” was characterized by a high intake of fresh and dried mushrooms, salted and dried vegetables, bean sprouts, ladies’ fingers, and other legumes. The second pattern, “high salt and sugar food,” consisted of lollies, ice cream, cakes and pastries, salted eggs, and sweet spreads. The third pattern, “processed food,” included processed chicken and meat products, packaged snacks and kuih, burgers, fried chicken, pizza, fries, mashed potatoes, coleslaw, and processed fish and seafood products. The fourth pattern, “nutritious food and dairy,” was characterized by a high intake of fruits, brown rice, cereals, grains, corn, yogurt, cheese, evaporated milk, and dried fruits.

**Table 3:** Rotated component matrix for dietary pattern (*N* = 181)

Food item	Dietary pattern			
	Plant-based food	High salt and sugar food	Processed food	Nutritious food and Dairy
Fresh & dried mushroom	0.735			
Salted & dried vegetables	0.725			
Bean sprout	0.706			
Ladies finger	0.621			
Other types of legumes	0.597			
Lollies & ice-cream		0.846		
Cake & pastry		0.819		
Salted egg		0.643		
Sweet spreads		0.476		
Processed chicken & meat products			0.822	
Packaged snacks & kuih*			0.611	
Burger, fried chicken, pizza, fries, mashed potatoes, coleslaw			0.599	
Processed fish, seafood products			0.575	
Fruits				0.709
Brown rice, cereal, grains, corn				0.586
Yogurt, cheese, evaporated milk				0.544
Dried fruits				0.541

*sweet and savory treats made from rice flour, coconut milk, and other ingredients

In a multivariate analysis (Table 4), age was positively associated with *H. pylori* seropositivity (OR: 1.042, 95% CI: 1.011–1.074), indicating a 4.2% increase in risk for every one-year increase in age. Conversely, systolic blood pressure was found to be negatively associated with the bacterium’s serostatus (OR: 0.967, 95% CI: 0.941–0.994). Regarding lifestyle factors, a high physical activity level acted as a protective factor, reducing the odds of seropositivity by 72.2% (OR: 0.278, 95% CI: 0.121–0.640) compared to low physical activity. Interestingly, a medium intake of nutritious food and dairy was associated with a significantly higher risk of seropositivity (OR: 3.364, 95% CI: 1.394–8.117) compared to low intake.

**Table 4:** Multivariate analysis of factors associated with *Helicobacter pylori* seropositivity (*N* = 181)

Variable	Odd ratio	95% CI	*p* value
Age	1.042	1.011–1.074	0.007
Systolic blood pressure	0.967	0.941–0.994	0.017
Physical activity level			
Low	reference		
High	0.278	0.121–0.640	0.003
Nutritious food & Dairy			
Low intake	reference		
Medium intake	3.364	1.394–8.117	0.007

## Discussion

To the best of our knowledge, this is the first community-based study conducted in Sarawak since the 2004 study on the Penan communities. In our study among the Dayak communities, we found a seroprevalence of 46.2%, a figure similar to those reported among Chinese communities in other local studies, but lower than that observed in Indian and other ethnic groups in Sarawak. Nevertheless, this prevalence is higher than that reported in the study on the Penan indigenous group. However, these comparisons must be interpreted with caution due to the different diagnostic modalities employed. Our study used a blood test to detect *H. pylori* antibodies, which indicate past or current exposure to the bacterium. Conversely, the Penan study determined prevalence using stool antigen detection, a method that identifies active, current infection. Because antibodies can persist in the bloodstream for months or years after an infection has been cleared, serology-based prevalence likely overestimates the burden of active infection compared to antigen detection.

The multivariate analysis revealed that age was positively associated with *H. pylori* seropositivity (OR: 1.042). This finding is consistent with previous epidemiological studies reporting a worldwide increase in *H. pylori* prevalence with age, with 40–60% of asymptomatic individuals among those who test positive [25], and as high as 70–85% among institutionalized older adults [34, 35]. The mechanism of exposure and subsequent seroconversion is complex and dynamic. According to Araujo et al. [36], *H. pylori* infection often starts in childhood and usually goes unnoticed because the immune response in children is predominantly regulatory (Treg), with higher levels of TGF-β1 and IL-10, and more FOXP3 + Treg cells in the gastric mucosa. This allows the bacteria to persist in the stomach with only mild inflammation and little damage. As people enter adulthood, the immune response shifts to a more inflammatory pattern, dominated by Th1 and Th17 cells. Adults have higher levels of IFN-γ, IL-12p70, IL-17 A, and IL-23, and lower levels of TGF-β1. This change leads to more intense inflammation and increases the risk of gastric damage, such as peptic ulcers, gastric atrophy, and intestinal metaplasia, especially in older adults [37, 38]. This may explain why the prevalence of *H. pylori* infection increases with age, likely due to cumulative exposure over time and the persistence of infection acquired during childhood, making older individuals more likely to be colonized by *H. pylori* compared to younger age groups.

Our study found that systolic blood pressure was significantly lower in participants with serological evidence of *H. pylori* exposure. This result contrasts with the findings of Kopacova et al. [39] and Xiong et al. [40], both of whom reported that *H. pylori* infection was associated with significantly higher blood pressure, particularly in older adults. This unexpected inverse association in our cohort requires cautious interpretation and should not be viewed as a protective effect of the infection.

One plausible explanation for this finding is reverse causation: individuals with symptomatic gastric distress related to *H. pylori* exposure may experience dyspepsia, leading to reduced appetite, weight loss, or a voluntary decrease in salt intake, all of which can lower blood pressure. Furthermore, residual confounding may be present; unmeasured factors such as the use of antihypertensive medications or differences in socioeconomic status could influence both infection risk and blood pressure levels.

Despite our observed results, the biological link between *H. pylori* and vascular damage is well-supported by recent molecular studies. Chronic infection can trigger systemic inflammation, characterized by elevated C-reactive protein and white blood cell counts [41, 42]. Beyond general inflammation, specific virulence factors—most notably the Cytotoxin-associated gene A (CagA) protein—have been shown to directly accelerate vascular endothelial dysfunction [43]. Recent evidence indicates that transgenically expressed CagA can promote arteriosclerosis, while exosomal CagA can induce macrophage foam cell formation, a critical stage in plaque development [44].

Additionally, the role of dietary salt must be considered, as high salt consumption is a known risk factor for both hypertension and increased *H. pylori* colonization and virulence. The discrepancy between our findings and existing literature may therefore reflect community-specific lifestyle factors, such as local salt intake patterns or a higher prevalence of less virulent (CagA-negative) strains in this population. Ultimately, the relationship between *H. pylori* and cardiovascular health is multifactorial and warrants further longitudinal investigation.

While some studies, such as Cheng et al. [45], suggest that *H. pylori* infection is not directly associated with physical activity, our findings showed a strong protective effect of high physical activity (OR = 0.278). This association should be interpreted with caution as physical activity may serve as a proxy for socioeconomic status (SES) or occupational exposure within the Dayak communities. In this rural setting, individuals with high physical activity levels are often engaged in active manual labor or traditional farming, which may be associated with different environmental exposures compared to those with more sedentary lifestyles. Furthermore, higher physical activity might correlate with better overall health literacy or higher SES in certain sub-groups, which in turn influences hygiene practices and access to clean water. While physical activity may theoretically reduce risk by enhancing immune function or regulating gastric acid secretion [46], it is likely that in this population, it reflects a complex interplay of multifactorial socio-economic and lifestyle factors. Future studies should include more granular data on occupation and household income to further disentangle these relationships.

The association between a medium intake of “nutritious food and dairy” and *H. pylori* seropositivity warrants a cautious, multi-faceted interpretation. While our data clearly demonstrate a statistically significant risk in this group (OR = 3.364), the reasons for this non-linear association remain hypothetical and require further investigation. This pattern may be attributed to a “healthy survivor” effect or differences in dietary sourcing and hygiene practices between groups. We hypothesize that individuals with a “high” intake of these items might possess higher socioeconomic status or better access to pasteurized dairy products compared to those with “medium” intake. Conversely, those with “medium” intake might rely on local, unpasteurized, or contaminated sources intermittently. In this context, the observed association may not be a direct effect of the food items themselves, but rather a reflection of the hygiene and safety of their preparation and sourcing. The possibility of food acting as a transmission vehicle is supported by broader epidemiological literature, although this was not directly measured in the current study. While many studies indicate that milk components like Lactobacillus and lactoferrin possess anti-inflammatory activity [47, 48, 49], we suggest that in rural settings, the risk of contamination may outweigh these nutritional benefits. For instance, milk and dairy products can serve as a vehicle for *H. pylori* transmission via contamination during milking from feces, soil, or water [50]. This is further supported by Momtaz et al. [51], who identified raw milk as a likely source of human infection. While our study did not test food samples for *H. pylori* DNA, these established transmission pathways provide a plausible theoretical framework for our findings.

Several limitations should be noted. First, due to the cross-sectional study design, we cannot determine causal relationships between various factors and *H. pylori* seropositivity. Second, data collection using survey methods may have introduced response bias, including recall bias in the dietary assessment using the food frequency questionnaire. Third, *H. pylori* seropositivity status was determined using *H. pylori*-specific IgG antibody, which may reflect both past and current infection. Fourth, the sample size was logistically constrained to 199 participants, resulting in a margin of error of approximately 7% based on a 37.5% expected prevalence; however, this provides adequate power for this exploratory study. Finally, the participation of only six out of ten randomly selected villages introduced potential selection bias. However, the non-participation of the four villages was due to logistical constraints related to community activities rather than systematic differences in health behaviors or environmental factors, and participating villages were geographically dispersed across the Kuching Division, maintaining the relevance of the findings.

## Conclusion

The findings of this study highlight that *H. pylori* exposure within the Dayak community is likely influenced by a complex interplay of factors, including age, physical activity, and specific dietary patterns. With a seroprevalence of 46.2% reflecting cumulative lifetime exposure, these results suggest that *H. pylori* represents a noteworthy public health consideration in this population. However, given the limitations of serological testing in distinguishing active from past infections, these findings should be viewed as a preliminary baseline of the community’s exposure history rather than a definitive measure of active clinical cases. 

To build upon these findings, local health authorities might consider exploring targeted surveillance strategies tailored to rural infrastructure. For instance, investigating the impact of strengthening the monitoring of gravity-fed water systems and communal storage tanks in rural longhouses could provide further insight into transmission dynamics. Furthermore, future public health initiatives could evaluate the feasibility of introducing point-of-care (POC) stool antigen screening within local health clinics. Such an exploratory approach would help determine the viability of a confirmed “test-and-treat” pathway for symptomatic patients in remote settings where invasive diagnostics are inaccessible. Ultimately, these findings serve as a hypothesis-generating foundation for future longitudinal research aimed at characterizing the active disease burden and long-term gastric health of rural indigenous populations.

## Data Availability

The datasets used and/or analyzed during the current study available from the corresponding author on reasonable request, with the permission of the funder.

## References

[1] Kao CHY, Sheu BS, Wu JJ. *Helicobacter pylori* infection: an overview of bacterial virulence factors and pathogenesis. Biomed J. 2016;39:14–23. 27105595 10.1016/j.bj.2015.06.002 PMC6138426

[2] Namikawa K, Purisevic FL, Thorsteinsson JB, Bjornsson ES. Helicobacter pylori Across Continents: Contrasts in Epidemiology, Genetics, Clinical Impact, and Management Between East and West. Int J Mol Sci. 2025;26:11408. 10.3390/ijms262311408. 41373564 10.3390/ijms262311408 PMC12691815

[3] Gantuya B, Bolor D, Oyuntsetseg K, Erdene-Ochir Y, Sanduijav R, Davaadorj D, Tserentogtokh T, Azzaya D, Uchida T, Matsuhisa T, et al. New observations regarding *Helicobacter pylori* and gastric cancer in Mongolia. Helicobacter. 2018;23:e12491. 29882322 10.1111/hel.12491 PMC6039254

[4] Hooi JKYm Lai WY, Ng WK, Suen MMY, Underwood FE, Tanyingoh D, Malfertheiner P, Graham DY, Wong VWS, Wu JCY, et al. Global Prevalence of *Helicobacter pylori* Infection: Systematic Review and Meta-Analysis. Gastroenterology. 2017;153:420–9. 28456631 10.1053/j.gastro.2017.04.022

[5] Ueda J, Gosho M, Inui Y, Matsuda T, Sakakibara M, Mabe K, Nakajima S, Shimoyama T, Yasuda M, Kawai T, et al. Prevalence of *Helicobacter pylori* infection by birth year and geographic area in Japan. Helicobacter. 2014;19:105–10. 24506211 10.1111/hel.12110

[6] Wang C, Nishiyama T, Kikuchi S, Inoue M, Sawada N, Tsugane S, Lin Y. Changing trends in the prevalence of *H. pylori* infection in Japan (1908–2003): A systematic review and meta-regression analysis of 170,752 individuals. Sci Rep 7. 2017 201;7:15491. 10.1038/s41598-017-15490-7. 10.1038/s41598-017-15490-7 PMC5686167 29138514

[7] Shah SC, Halvorson AE, Lee D, Bustamante R, McBay B, Gupta R, Denton J, Dorn C, Wilson O, Peek R Jr., et al. *Helicobacter pylori* Burden in the United States According to Individual Demographics and Geography: A Nationwide Analysis of the Veterans Healthcare System. Clin Gastroenterol Hepatol. 2024;22:e4226–5026. 10.1016/j.cgh.2023.05.016 PMC11552088 37245717

[8] Sugano K, Tack J, Kuipers EJ, Graham DY, El-Omar EM, Miura S, Haruma K et al. faculty members of Kyoto Global Consensus Conference. Kyoto global consensus report on *Helicobacter pylori* gastritis. Gut. 2015; 64: 1353–1367 [PMID: 26187502 10.1136/gutjnl-2015-309252] 10.1136/gutjnl-2015-309252 PMC4552923 26187502

[9] Malfertheiner P, Megraud F, O’Morain CA, Gisbert JP, Kuipers EJ, Axon AT, et al. European *Helicobacter* and Microbiota Study Group and Consensus panel. Management of Helicobacter pylori infection-the Maastricht V/Florence Consensus Report. Gut. 2017;66:6–30. 10.1136/gutjnl-2016-312288]. [PMID:27707777. 27707777 10.1136/gutjnl-2016-312288

[10] Zhang F, Pu K, Wu Z, Zhang Z, Liu X, Chen Z, et al. Prevalence and associated risk factors of *Helicobacter pylori* infection in the Wuwei cohort of north-western China. Trop Med Int Health. 2021;26:290–300. 10.1111/tmi.13517]. [PMID: 33159827. 33159827 10.1111/tmi.13517

[11] Vandenplas Y. *Helicobacter pylori* infection. Clin Microbiol Infect. 1999;5:1–11. 11856206

[12] Wroblewski LE, Peek RM Jr, Wilson KT. *Helicobacter pylori* and gastric cancer: factors that modulate disease risk. Clin Microbiol Rev. 2010;23(4):713–39. 10.1128/CMR.00011-10. PMID: 20930071; PMCID: PMC2952980. 20930071 10.1128/CMR.00011-10 PMC2952980

[13] Mousavi S, Ilaghi M, Elahi Vahed I, et al. Epidemiology and socioeconomic correlates of gastric cancer in Asia: results from the GLOBOCAN 2020 data and projections from 2020 to 2040. Sci Rep. 2025;15:6529. 10.1038/s41598-025-90064-6. 39988724 10.1038/s41598-025-90064-6 PMC11847935

[14] Goh KL. Lessons learnt from the epidemiology of *Helicobacter pylori* infection in Malaysia: JGHF Marshall and Warren Lecture 2017. Journal of Gastroenterology and Hepatology. 2018; 33 (2018) 1177–1184. 10.1111/jgh.14131 29498759

[15] Goh KL. The racial cohort phenomenon: sero-epidemiology of *Helicobacter pylori* infection in a multiracial South-East Asian country. Eur J Gastroenterol Hepatol. 2003;13:177–83. 10.1097/00042737-200102000-00014 11246618

[16] Huang SS, Hassan AK, Choo KE, Ibrahim MI, Davis TM. Prevalence and predictors of *Helicobacter pylori* infection in children and adults from the Penan ethnic minority of Malaysian Borneo. Am J Trop Med Hyg. 2004;71:444–50. 15516641

[17] Ding SZ, Du YQ, Lu H, Wang WH, Cheng H, Chen SY, et al. National Clinical Research Center for Digestive Diseases (Shanghai), Gastrointestinal Early Cancer Prevention & Treatment Alliance of China (GECA), *Helicobacter pylori* Study Group of Chinese Society of Gastroenterology, and Chinese Alliance for Helicobacter pylori Study. Chinese Consensus Report on Family-Based Helicobacter pylori Infection Control and Management (2021 Edition). Gut. 2022;71:238–53. 10.1136/gutjnl-2021-325630]. [PMID: 34836916. 34836916 10.1136/gutjnl-2021-325630 PMC8762011

[18] Perry S, de la Luz Sanchez M, Yang S, Haggerty TD, Hurst P, Perez-Perez G, et al. Gastroenteritis and transmission of *Helicobacter pylori* infection in households. Emerg Infect Dis. 2006;12:1701–8. 10.3201/eid1211.060086]. [PMID: 17283620. 17283620 10.3201/eid1211.060086 PMC3372328

[19] Gunji T, Matsuhashi N, Sato H, et al. *Helicobacter pylori* infection is significantly associated with metabolic syndrome in the Japanese population. Am J Gastroenterol. 2008;103(12):3005–10. 19086952 10.1111/j.1572-0241.2008.02151.x

[20] Saijo Y, Utsugi M, Yoshioka E, et al. Relationship of *Helicobacter pylori* infection to arterial stiffness in Japanese subjects. Hypertens Res. 2005;28(4):283–92. 16138557 10.1291/hypres.28.283

[21] Xu C, Yan M, Sun Y, et al. Prevalence of *Helicobacter pylori* infection and its relation with body mass index in a Chinese population. Helicobacter. 2014;19(6):437–42. 25256639 10.1111/hel.12153

[22] Henriksson AE, Edman AC, Nilsson I, Bergqvist D, Wadstrom T. *Helicobacter pylori* and the relation to other risk factors in patients with acute bleeding peptic ulcer. Scand J Gastroenterol. 1998;33:1030–3. 9829355 10.1080/003655298750026705

[23] Everhart JE, Byrd-Holt D, Sonnenberg A. Incidence and risk factors for self-reported peptic ulcer disease in the United States. Am J Epidemiol. 1998;147:529–36. 9521179 10.1093/oxfordjournals.aje.a009484

[24] Fox JG, Dangler CA, Taylor NS, King A, Koh TJ, Wang TC. High-salt diet induces gastric epithelial hyperplasia and parietal cell loss, and enhances *Helicobacter pylori* colonization in C57BL/6 mice. Cancer Res. 1999;59(19):4823–8. [PubMed ID: 10519391]. 10519391

[25] Tsugane S, Tei Y, Takahashi T, Watanabe S, Sugano K. Salty food intake and risk of *Helicobacter pylori* infection. Jpn J Cancer Res. 1994;85(5):474–8. 10.1111/j.1349-7006.1994.tb02382.x. [PubMed ID: 8014104]. [PubMed Central ID:PMC5919501]. 8014104 10.1111/j.1349-7006.1994.tb02382.x PMC5919501

[26] Xia Y, Meng G, Zhang Q, Liu L, Wu H, Shi H, et al. Dietary Patterns are Associated with *Helicobacter Pylori* Infection in Chinese Adults: A Cross-Sectional Study. Sci Rep. 2016;6:32334. 10.1038/srep32334. PMID: 27573193; PMCID: PMC5004161. 27573193 10.1038/srep32334 PMC5004161

[27] Shu L, Zheng PF, Zhang XY, Feng YL. Dietary patterns and *Helicobacter pylori* infection in a group of Chinese adults ages between 45 and 59 years old: An observational study. Med (Baltim). 2019;98(2):e14113. PMID: 30633225; PMCID: PMC6336658. 10.1097/MD.0000000000014113 PMC6336658 30633225

[28] Mahendran H, Siow SL, Hardin Mark, Hashimah A, Azim N. Epidemiology of *Helicobacter pylori* infection in the island of Borneo. J Gastroenterol Hepatol. 2012;27:23–23.

[29] Institute for Public Health (IPH). National Health and Morbidity Survey 2014: Malaysian Adult Nutrition Survey. Vol. 1: Methodology and General Findings: 108 pages. 2014.

[30] Hosmer DW, Lemeshow S, Sturdivant RX. Applied Logistic Regression. 3rd ed. Wiley; 2013.

[31] Yong AG, Pearce S. A beginner’s guide to factor analysis: focusing on exploratory factor analysis. Tutorials Quant Methods Psychol. 2013;9(2):79–94. 10.20982/tqmp. 09.2. p079.

[32] Institute for Public Health (IPH), National Institutes of Health, Ministry of Health Malaysia. National Health and Morbidity Survey (NHMS) 2019: Vol. I: NCDs - Non-Communicable Diseases. Risk Factors and other Health Problems; 2020.

[33] Pilotto A, Salles N. *Helicobacter pylori* infection in geriatrics. Helicobacter. 2002;7(Suppl 1):56–62. 10.1046/j.1523-5378.7.s1.9.x]. [PMID: 12197911. 12197911 10.1046/j.1523-5378.7.s1.9.x

[34] Regev A, Fraser GM, Braun M, Maoz E, Leibovici L, Niv Y. Seroprevalence of *Helicobacter pylori* and length of stay in a nursing home. Helicobacter. 1999;4:89–93. 10.1046/j.1523-5378.1999.98640.x]. [PMID: 10382121. 10382121 10.1046/j.1523-5378.1999.98640.x

[35] Pilotto A, Fabrello R, Franceschi M, Scagnelli M, Soffiati F, Di Mario F, et al. *Helicobacter pylori* infection in asymptomatic elderly subjects living at home or in a nursing home: effects on gastric function and nutritional status. Age Ageing. 1996;25:245–9. 10.1093/ageing/25.3.245]. [PMID: 8670562. 8670562 10.1093/ageing/25.3.245

[36] Araújo GRL, Marques HS, Santos MLC, da Silva FAF, da Brito BB, Correa Santos GL, et al. *Helicobacter pylori* infection: How does age influence the inflammatory pattern? World J Gastroenterol. 2022;28(4):402–11. 35125826 10.3748/wjg.v28.i4.402 PMC8790560

[37] Freire de Melo F, Rocha AM, Rocha GA, Pedroso SH, de Assis Batista S, Fonseca de Castro LP, Carvalho SD, et al. A regulatory instead of an IL-17 T response predominates in *Helicobacter pylori*-associated gastritis in children. Microbes Infect. 2012;14:341–7. 10.1016/j.micinf.2011.11.008]. [PMID: 22155622. 22155622 10.1016/j.micinf.2011.11.008

[38] Razavi A, Bagheri N, Azadegan-Dehkordi F, Shirzad M, Rahimian G, Rafieian-Kopaei M, et al. Comparative Immune Response in Children and Adults with H. pylori Infection. J Immunol Res. 2015;2015:315957. 10.1155/2015/315957. PMID: 26495322. 26495322 10.1155/2015/315957 PMC4606101

[39] Kopácová M, Bures J, Koupil I, Rejchrt S, Vorísek V, Seifert B, European Society for Primary Care Gastroenterology, et al. Body indices and basic vital signs in *Helicobacter pylori* positive and negative persons. Eur J Epidemiol. 2007;22(1):67–75. 10.1007/s10654-006-9090-1. Epub 2006 Dec 29. PMID: 17195049; PMCID: PMC2799154. 17195049 10.1007/s10654-006-9090-1 PMC2799154

[40] Xiong X, Chen J, He M, Wu T, Yang H. *Helicobacter pylori* infection and the prevalence of hypertension in Chinese adults: The Dongfeng-Tongji cohort. J Clin Hypertens (Greenwich). 2020;22(8):1389–95. 10.1111/jch.13928. Epub 2020 Jul 20. PMID: 32687255; PMCID: PMC8030092. 32687255 10.1111/jch.13928 PMC8030092

[41] Kiechl S, Egger G, Mayr M, et al. Increased risk of atherosclerosis is confined to CagA-positive Helicobacter pylori strains: prospective results from the Bruneck Study. Stroke. 2001;32(9):2062–9. 10.1161/01.STR.0000058481.82639.EF 12624280

[42] Xie J, Jia L, Liu J, et al. Exosomal CagA derived from Helicobacter pylori-infected gastric epithelial cells induces macrophage foam cell formation and promotes atherosclerosis. J Mol Med. 2020;98(10):1411–23. 10.1016/j.yjmcc.2019.07.011 31352044

[43] Sugimoto M, Murata M, Itoi T, et al. Transgenically expressed Helicobacter pylori CagA in vascular endothelial cells accelerates arteriosclerosis in mice. Sci Rep. 2022;12(1):1–12. 35716599 10.1016/j.bbrc.2022.06.010

[44] Gancz H, Jones KR, Merrell DS. Helicobacter pylori responses to low iron and high salt: focus on transcription and virulence. Front Microbiol. 2020;11:130.32180763

[45] Cheng Y, Macera CA, Davis DR, Blair SN. Physical activity and peptic ulcers. Does physical activity reduce the risk of developing peptic ulcers? West J Med. 2000;173(2):101–7. 10.1136/ewjm.173.2.101. PMID: 10924430; PMCID: PMC1071012. 10924430 10.1136/ewjm.173.2.101 PMC1071012

[46] Everhart JE, Byrd-Holt D, Sonnenberg A. Incidence and risk factors for self-reported peptic ulcer disease in the United States. Am J Epidemiol. 1998;147:529–36. 9521179 10.1093/oxfordjournals.aje.a009484

[47] Felley C, Michetti P. Probiotics and *Helicobacter pylori*. Best Pract Res Clin Gastroenterol. 2003;17:785–91. 10.1016/s1521-6918(03)00070-2. 14507588 10.1016/s1521-6918(03)00070-2

[48] Gill HS. Probiotics to enhance anti-infective defences in the gastrointestinal tract. Best Pract Res Clin Gastroenterol. 2003;17:755–73. 10.1016/s1521-6918(03)00074-x. 14507586 10.1016/s1521-6918(03)00074-x

[49] Dial EJ, Hall LR, Serna H, Romero JJ, Fox JG, Lictenbergerger LM. Antibiotic properties of bovine lactoferrin on *Helicobacter pylori*. J Clin Microbiol. 1998;34:2593–4. 10.1023/a:1026675916421. 10.1023/a:1026675916421 9881510

[50] Chatterjee A, Yasmin T, Bagchi D, Stohs SJ. Inhibition of *Helicobacter pylori* in vitro by various berry extracts, with enhanced susceptibility to clarithromycin. Mol Cell Biochem. 2004;265:19–26. 10.1023/b:mcbi.0000044310.92444.ec. 15543930 10.1023/b:mcbi.0000044310.92444.ec

